# 3D printing of foetal vascular rings: feasibility and applicability

**DOI:** 10.1186/s12884-023-05683-6

**Published:** 2023-05-16

**Authors:** Jia Huang, Hao Wang, Yuanting Yang, Qian Chen, Jiaqi Hu, Hua Shi, Qing Zhou

**Affiliations:** 1 Department of Obstetrics and Gynecology Ultrasound, Renmin Hospital of Wuhan University, Wuhan, 430060 China; 2 Department of Ultrasound Imaging, Renmin Hospital of Wuhan University, Wuhan, 430060 China

**Keywords:** Echocardiography, 3D printing, Fetus, Vascular rings

## Abstract

**Background:**

Vascular rings (VRs) exhibit complex and diverse forms that are difficult to conceptualize using traditional two-dimensional (2D) schematic. Inexperienced medical students and parents who lack a medical technology background face significant challenges in understanding VRs. The purpose of this research is to develop three-dimensional (3D) printing models of VRs to provide new technical imaging support for medical education and parental consultation.

**Methods:**

This study included 42 fetuses diagnosed as VRs. Foetal echocardiography, modeling and 3D printing were performed, and the dimensional accuracy of models was analyzed. The value of 3D printing in the teaching of VRs was analyzed based on comparing the test results before and after the teaching intervention of 48 medical students and the satisfaction survey. A brief survey was conducted to 40 parents to assess the value of the 3D printed model in prenatal consultations.

**Results:**

Forty models of VRs were successfully obtained, which reproduced the anatomical shape of the VRs space with high dimensional accuracy. No differences in the prelecture test results were noted between the 3D printing group and the 2D image group. After the lecture, the knowledge of both groups improved, but the postlecture score and the change in the prelecture versus postlecture score were greater in the 3D printing group, and the subjective satisfaction survey feedback in the 3D printing group was also better (*P* < 0.05). Similar results were observed from the parental questionnaire, the vast majority of parents have an enthusiastic and positive attitude towards the use of 3D printed models and suggest using them in future prenatal consultations.

**Conclusions:**

Three-dimensional printing technology providing a new tool for effectively displaying different types of foetal VRs. This tool helps physicians and families understand the complex structure of foetal great vessels, positively impacting medical instruction and prenatal counselling.

## Background

Vascular rings (VRs), once the subject of case reports [1], are now recognized as uncommon but not exceedingly rare cardiovascular malformations [2, 3]. Prognosis varies widely among patients with different types of VRs, and certain types (e.g., double aortic arch or pulmonary artery sling) could cause serious clinical symptoms associated with tracheal and/or oesophageal compression, including stridor, dysphagia and recurrent respiratory infections, which could occur in early infancy [3]. Most infants present feeding issues or airway obstruction in their first year of life [4]. Thus, it is necessary to confirm the type of VRs prenatally and conduct family counselling to develop prospective care plans.

Currently, prenatal ultrasound is the preferred method to diagnose VRs and relies heavily on the technical skills of the operator. Effective training is essential to ensure consistently high diagnostic rates and quality care after birth. However, vascular anatomy is difficult to conceptualize using traditional two-dimensional (2D) schematics, and many medical students still have insufficient understanding of the classification of VRs given the presence of complex and diverse abnormal forms. Parents, who typically lack this level of technical background, face an even greater challenge in attempting to understand the specifics of their children’s cardiovascular malformations [5]. In addition, it is also very difficult to interpret the spatial relationship from sequential 2D views because the complex VR structures typically involve multiple vessel abnormalities.

Three-dimensional ultrasound (3DUS) enables a more detailed assessment of the embryo and the foetus compared with 2D ultrasound [6]. However, 3DUS involves visualization on a flat screen, which limits the interpretation of complex spatial structures [7]. The introduction and development of 3D printing technology makes it possible to use hand-held individual anatomical models for teaching purposes and family counselling. 3D printing technology has been used in cases of foetal congenital anomalies for presurgical planning in obstetrics [8]; didactic and research purposes in other areas of medicine, e.g., 3D printing foetal brains [9]; and interactions with visually impaired pregnant patients [10]. In previous studies, our group initially validated the feasibility of constructing 3D models of the foetal heart and successfully produced a series of foetal heart models with abnormal ventricular-arterial connections [11]. In this study, we further focused on the branches of the great arteries in the upper mediastinum of the foetus, aiming to obtain different types of VR models. We hope to provide new technical imaging support for understanding the complexity of VRs and to systematically evaluate the feasibility and applicability of the use of 3D models to teach medical students about VRs and to provide effective prenatal counselling.

## Methods

### Study design

**Fig. 1:**
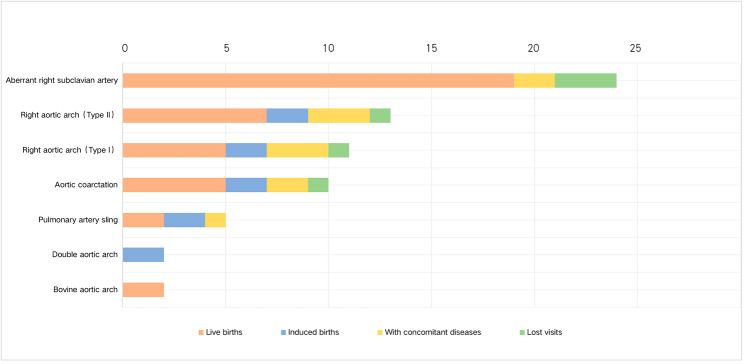
General information of the 42 foetuses included in this study

This study’s protocol was reviewed and approved by the Medical Ethics Committee of Renmin Hospital of Wuhan University (No. WDRY2018-K032). The safety and limitations of the examination were informed to the participants who gave their informed consent as required. Forty-two foetuses diagnosed with VRs and subject to cardiac ultrasound from May 2018 to December 2020 were enrolled in this study, with gestational weeks ranging from 21 to 31 weeks. Exclusion criteria comprised cases with poor 2D image quality, too much or little amniotic fluid, or foetal arrhythmia. The general information of the 42 cases of foetal aortic arch anomalies is provided in Fig. 1, and these cases were classified into the following 4 group based on the type of VR. The left aortic arch group comprised 21 cases, including 19 cases with an aberrant right subclavian artery and 2 cases with a bovine aortic arch. The right aortic arch group comprised 12 cases, including 5 cases with Type I anomalies (right arch with mirror-image branching) and 7 cases with Type II anomalies (right arch with aberrant left subclavian artery) [12]. The double aortic arch group comprised 2 cases. The other group comprised 7 cases, including 5 cases of aortic coarctation and 2 cases of pulmonary artery sling. All cases involved left-sided ductus arteriosus. Eleven cases were diagnosed with additional cardiac lesions, including ventricular septal defects and conotruncal heart defects.

### Volume data acquisition

The spatiotemporal image correlation (STIC) volume data for each case were obtained using Voluson E10 ultrasound system (GE Healthcare, Chicago, IL, USA) and GE RM6C ultrasound transducer at a frequency of 2–6 MHz. Maintain high frequency and scanning density machine setting, adjust the scanning angle to maintain the foetal spine at 5–7 o ‘clock. View of three-vessel trachea or coronal scan of the aortic arch was selected as the initial section at STIC mode after moderate magnification of the image. The angle was adjusted according to the gestational week of the foetus, with 20–30 degrees for mid-trimester and 35–45 degrees for late trimester. The volume acquisition lasted 12 s. It is worth noting that as much of the brachiocephalic arteries as possible should be shown during data collection. Foetal movement necessitated repeated volume acquisitions to obtain satisfactory data. At least 5 qualified volume data without obvious artifacts were acquired in each case for subsequent data analysis.

### 3D printing protocol

The volume data were exported as Cartesian.vol files and loaded into Mimics (version 19.0, Materialise, Leuven Belgium) for postprocessing. Threshold segmentation of the upper mediastinal region of interest was performed, during which the image boundaries were confirmed by an experienced sonographer. The minimum threshold was set to 0, and the maximum value was set to 65–110. Then, manual interactive segmentation was applied to remove noise signals and adjacent structures layer by layer. Objects were generated and exported to 3-MATIC (Version 11, Materialise, Leuven Belgium) using the following steps: repairing, wrapping and smoothing (the smoothing factor is 1.0) to reduce pixelation and any other errors/gaps in the model, and then adding a layer of surface with 0.5 mm thickness based on the vascular model. The 3D digital models of the vessel and its branches, including the ascending aorta, aortic arch, aortic arch branches, ductus arteriosus, descending aorta, and pulmonary artery, were generated and subsequently saved as standard tessellation language (STL)format files.

**Fig. 2:**
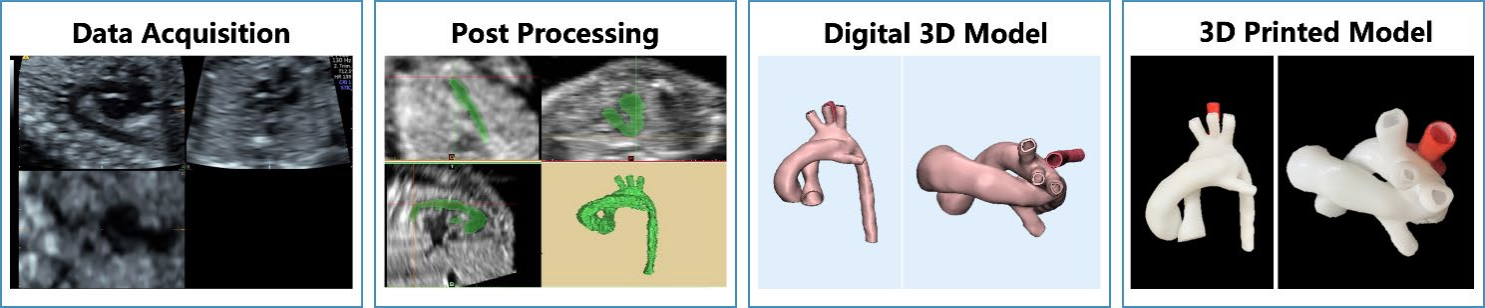
Workflow of 3D printing

The chosen STL files were printed with a Form2 3D printer (Formlabs, Boston, MA) using stereolithography printing technology in photosensitive resin material (Formlabs, Boston, MA), which is a white opaque, hard and inelastic material. All models were 1:1 in size and scaled with a 3-fold factor to provide a deeper view of such small-sized cardiovascular structures. For a clearer display of the relationship between blood vessels and the trachea, a 3D-printed model of the trachea and bronchus was also constructed for this study. The workflow of 3D printing is shown in Fig. 2.

### Quantitative assessment: analysis of 3D model accuracy

**Fig. 3:**
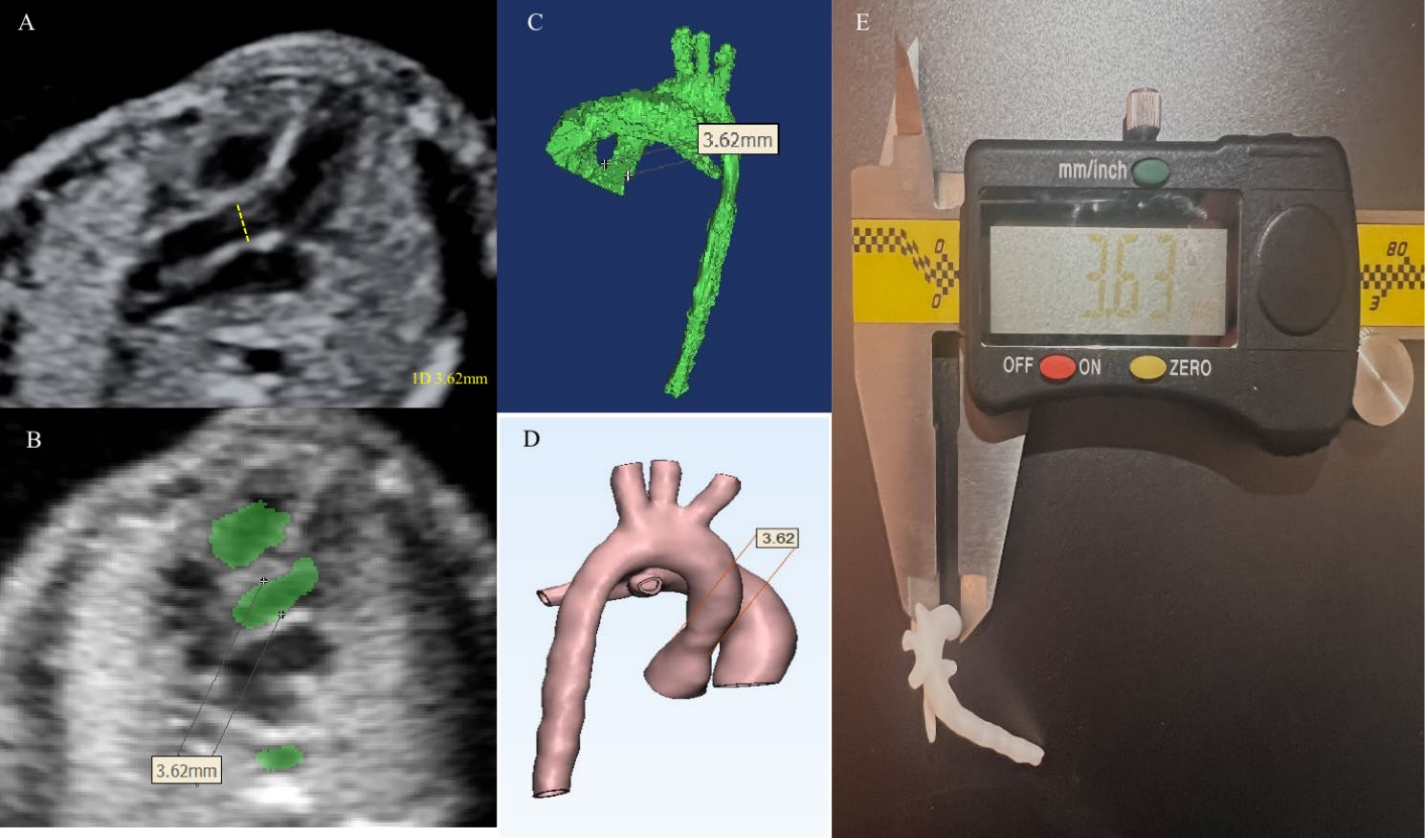
3D model accuracy evaluation. **A.** Aortic diameter measurement in the echocardiogram. **B.** STIC original data in the transection view to measure aortic diameter in Mimics. **C.** 3D object to measure aortic diameter in Mimics. **D.** 3D digital model to measure aortic diameter in the 3-MATIC. **E.** Measurement of the aortic diameter in the 3D printed model

A frame plane in the Mimics segmented data set was selected for measurement to ensure that the two-dimensional echocardiogram was the same as the plane on the segmented data set. Three anatomical sites were selected for each case based on the characteristics of the cases and the feasibility of the measurements. Measurements obtained using the ruler measurement tool included diameters of the aorta (AO), pulmonary artery (PA), ductus arteriae (DA), left pulmonary artery (LPA), and right pulmonary artery (RPA). After the measurement is completed, the corresponding measurement position and value can be displayed synchronously on the 3D digital model, and the accuracy of the corresponding position of the 1:1 scale 3D printing model can be measured with the vernier caliper, as shown in Fig. 3. Each anatomical location was measured three times with the mean value considered as final to reduce the bias. The accuracy of the 3D modeling was evaluated by comparing the difference of measurement between the model and image data.

### Qualitative assessment: applicability of 3D models

#### Medical education

Forty-eight ultrasound students from our hospital’s obstetrics and gynecology ultrasound department who received standardized training as resident physicians were recruited. Briefly, 48 third-year medical students were randomly assigned to either the 2D image group (n = 24) taught with the aid of a hand-drawn schematic diagram or the 3D printing group (n = 24) taught with the aid of 3D printed models. Both groups attended the same lecture on VRs in two separate 60-minute sessions each, presenting standard slides on embryology, anatomy, and classification as well as prenatal diagnosis and postnatal management of VRs. After an initial description of each category of VRs, both groups of students were allowed to freely analyse and manipulate teaching aids (2D hand-drawn schematics or 3D printed models) throughout the lecture. Students were also free to ask questions about the models or schematics during the lecture.

To evaluate knowledge acquisition, each student answered the same multiple-choice test twice, pre- and postlecture, with a maximal score of 100 points corresponding to 10 questions. The test assessed knowledge acquisition on embryology, anatomy, etc. At the end of the course, both groups were required to complete a brief 5-question rated on a 10-point Likert scale to establish the perceived effectiveness of the educational method to the student.

#### Parent questionnaire survey

After obtaining consent, a cardiologist explain the child’s heart condition to 40 parents using a 2D hand-drawn schematic diagram. Then, the parents received the same explanation using a 3D-printed heart model. After each consultation, parents received a short survey of 4 questions rated on a 5-point Likert scale to assess their perception of the use of 3D models in prenatal consultation. We deliberately chose to keep the questions concise and direct, as the scope of this study was limited to initial impressions of understanding based on each modality.

### Statistical analysis

Statistical analyses were performed using SPSS Version 22.0 (SPSS Inc., Chicago, IL). Continuous variables were described as the means ± standard deviations. Paired t test was used to compare the measurement results between ultrasound images and 3D models. To assess the agreement between ultrasound images and 3D models, a Bland‒Altman analysis (Version 22.0, SPSS Inc., Chicago, IL) was used. The increase in score from pre- to posttest was calculated as the difference in number of questions answered correctly. Normally distributed continuous variables were compared with t tests and non-normally distributed variables were compared with Mann-Whitney tests, and categorical variables were compared with Fisher’s exact test. Changes in subjective survey scores were compared with the Wilcoxon signed rank test. All tests were assessed at the P value < 0.05 level of significance.

## Results

### General information of the subjects

A total of 42 foetal cardiac STIC images meeting the inclusion requirements were included in this study, 2 cases were excluded from successful 3D printing post-processing due to poor image quality, and 40 fetuses were ultimately included. The success rate of postprocessing was 95% (40/42).

### General 3D printing information

**Fig. 4:**
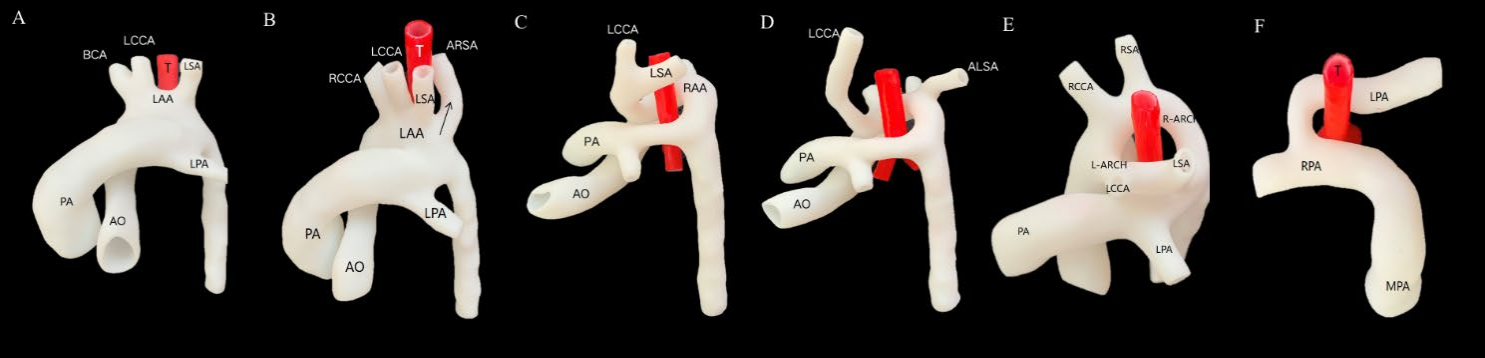
The 3D printing models of 6 cases. **A.** Bovine aortic arch. **B.** Aberrant right subclavian artery. **C.** Right aortic arch (type I). **D.** Right aortic arch (type II). **E.** Double aortic arch. **F.** Pulmonary artery sling. BCA: brachiocephalicus; LCCA: left common carotid artery; LSA: left subclavian artery; LAA/L-ARCH: left aortic arch; MPA/PA: pulmonary artery; DA: ductus arteriosus; RPA: right pulmonary artery; LPA: left pulmonary artery; T: trachea. RCCA: right common carotid artery; RSA: right subclavian artery; RAA/R-ARCH: right aortic arch; SVC: superior vena cava; DAO: descending aorta; AO: aorta; LSVC: left superior vena cava

The 3D model contained a wealth of spatial anatomical details, including the branches of the aortic arch or pulmonary artery. As shown in Fig. 4A, the 3D model of the bovine aortic arch clearly showed the common origin of the brachiocephalic artery and the left common carotid artery from the aortic arch. The 3D model of the left aortic arch with an aberrant right subclavian artery demonstrated that the fourth branch of the arch originated at the beginning of the descending aorta and extended to the right and behind the trachea (Fig. 4B). The type of right aortic arch could be determined by models showing the location of the aortic arch and the origin of the main branches (Fig. 4C and D). The 3D model of the double aortic arch showed that the ascending aorta was divided into the left and right aortic arch and converged into the descending aorta. Branches on the left and right aortic arch could also be observed (Fig. 4E). The 3D model of aortic coarctation displayed the abnormal diameter of the aortic isthmus, which can be visually compared to the surrounding vessels. The 3D model of the pulmonary sling showed that the left pulmonary artery originated from the right pulmonary artery (Fig. 4F).

It took approximately 4 h to produce the model (including image segmentation, postprocessing and printing). The total material cost of each 1:1 and 1:3 scale model material cost is approximately 10¥ and 30¥, respectively.

### Quantitative measurements of anatomical accuracy

**Fig. 5:**
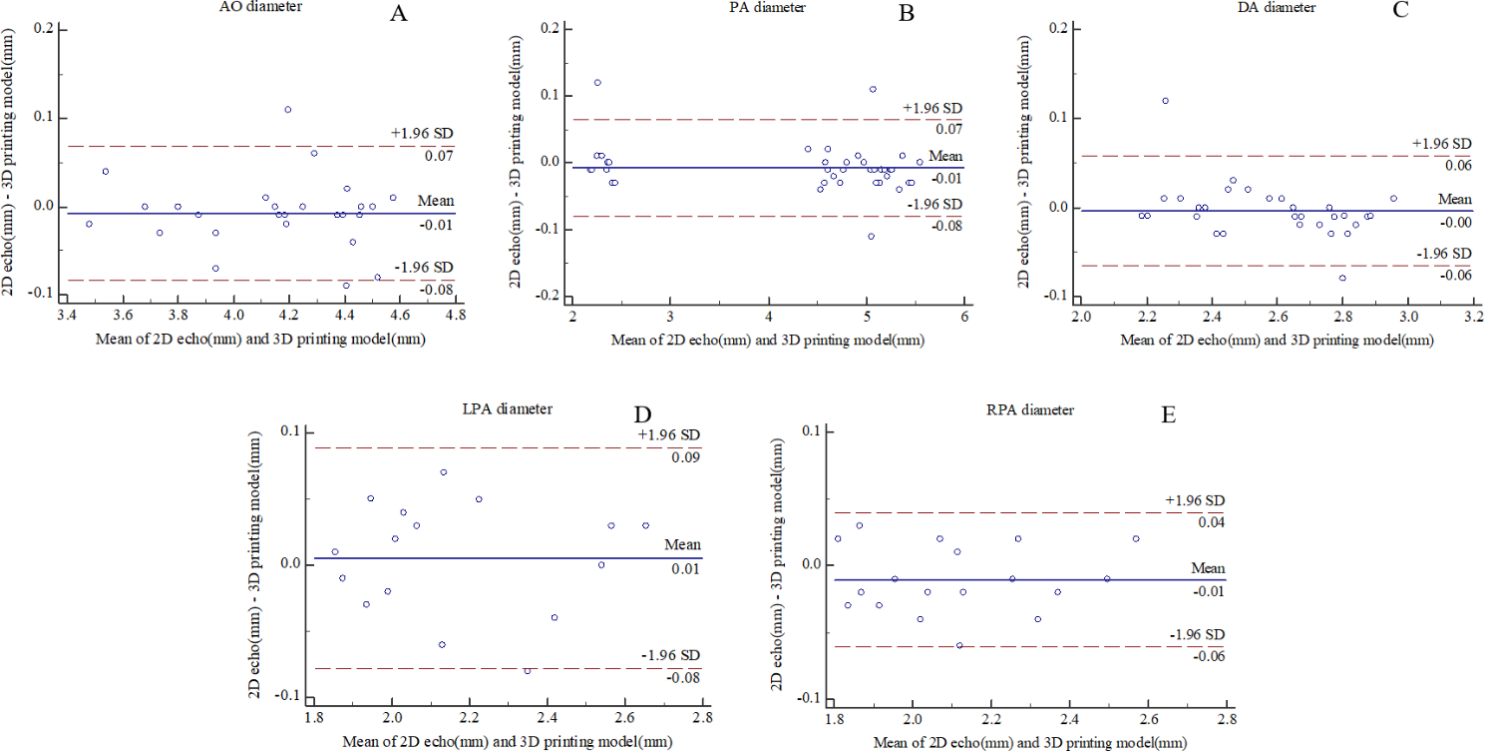
Bland–Altman plots show comparison of AO diameter **(A)**, PA diameter **(B)**, DA diameter **(C)**, LPA diameter **(D)** and RPA diameter **(E)** measurements between the 2D echo and 3D printing model. AO: aorta; PA: pulmonary artery; DA: ductus arteriosus; LPA: left pulmonary artery; RPA: right pulmonary artery; 3D: three-dimensional; SD: standard deviation

No significant differences in the parameters of the great artery (AO, PA, LPA, RPA, and DA) were noted between the printed models and echocardiographic images (P > 0.05). The Bland‒Altman scatter plots showed that of the five measurement sites, there are two data points (7.1%) of AO and PA diameter, two data points (6.7%) of DA diameter, one data points (6.2%) of LPA diameter, and one data points (5.6%) of RPA diameter were outside the consistency bound between 2D echo and 3D printing model measurements. The consistency bound were (-0.09 mm, 0.11 mm), (-0.11 mm, 0.11 mm), (-0.1 mm, 0.12 mm), (-0.08 mm, 0.07 mm), (-0.07 mm, 0.06 mm), respectively (Fig. 5).

### Questionnaire survey

**Fig. 6:**
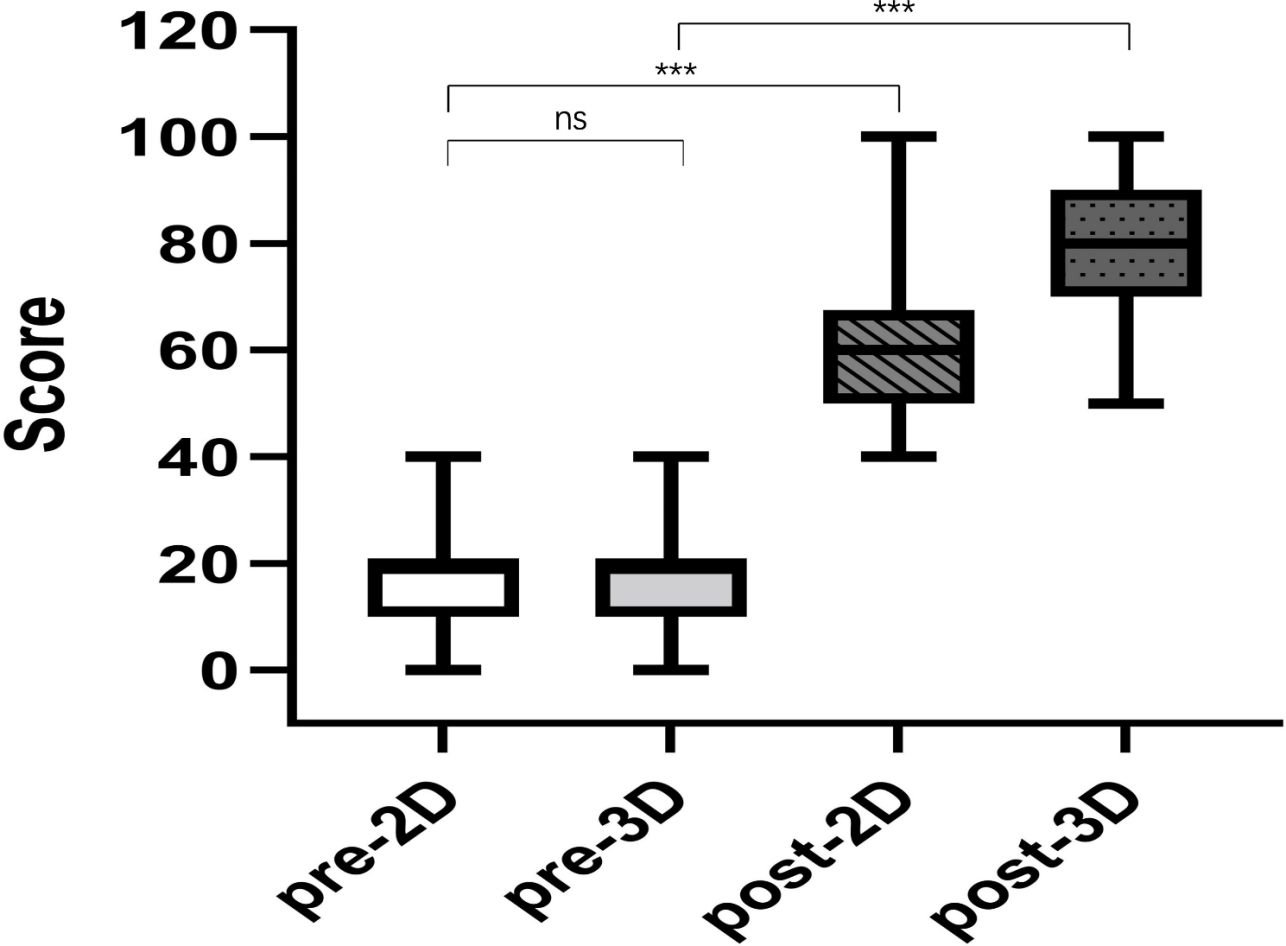
Pre-and postlecture test results for the 2D image and 3D printing groups, ***P < 0.001

Overall, no difference in prelecture test scores were noted between the 3D printing and 2D image groups. Median global scores of 19.58 ± 8.88 and 18.33 ± 8.8 were obtained by the 2D and 3D groups, respectively (*P* = 0.67). Knowledge improved in both groups after the lecture, but greater improvements were noted in the 3D group compared with the 2D group (77.08 ± 14.28 vs. 54.58 ± 13.65, *P* < 0.0001). A greater increase in the score was noted for the printing group compared with the control group (61.25 ± 16.4 vs. 39.17 ± 19.13, *P* = 0.001) (Fig. 6). The satisfaction among students in the 3D printing Group and 2D image Group is summarized in Table 1. In the postlecture subjective satisfaction survey, the 3D printing group provided more positive feedback regarding learning interest, learning efficiency, education depth, novelty and inspiration, and general satisfaction compared with the 2D group, and the difference was statistically significant (*P* < 0.05).

**Table 1:** Satisfaction among students in the 3D printing Group and 2D image Group

Appraisal (score)	3D printing Group	2D image Group	*P* Value	
Learning interest Learning efficiency	7.5 ± 0.7 8.3 ± 0.4	5.4 ± 0.5 6.1 ± 0.5	0.03 0.01	
Education depth	7.2 ± 0.8	5.6 ± 0.7	0.03	
Novel and inspiring	8.8 ± 0.6	5.2 ± 0.6	< 0.001	
General satisfaction	7.9 ± 0.5	5.7 ± 0.4	0.01	

**Fig. 7:**
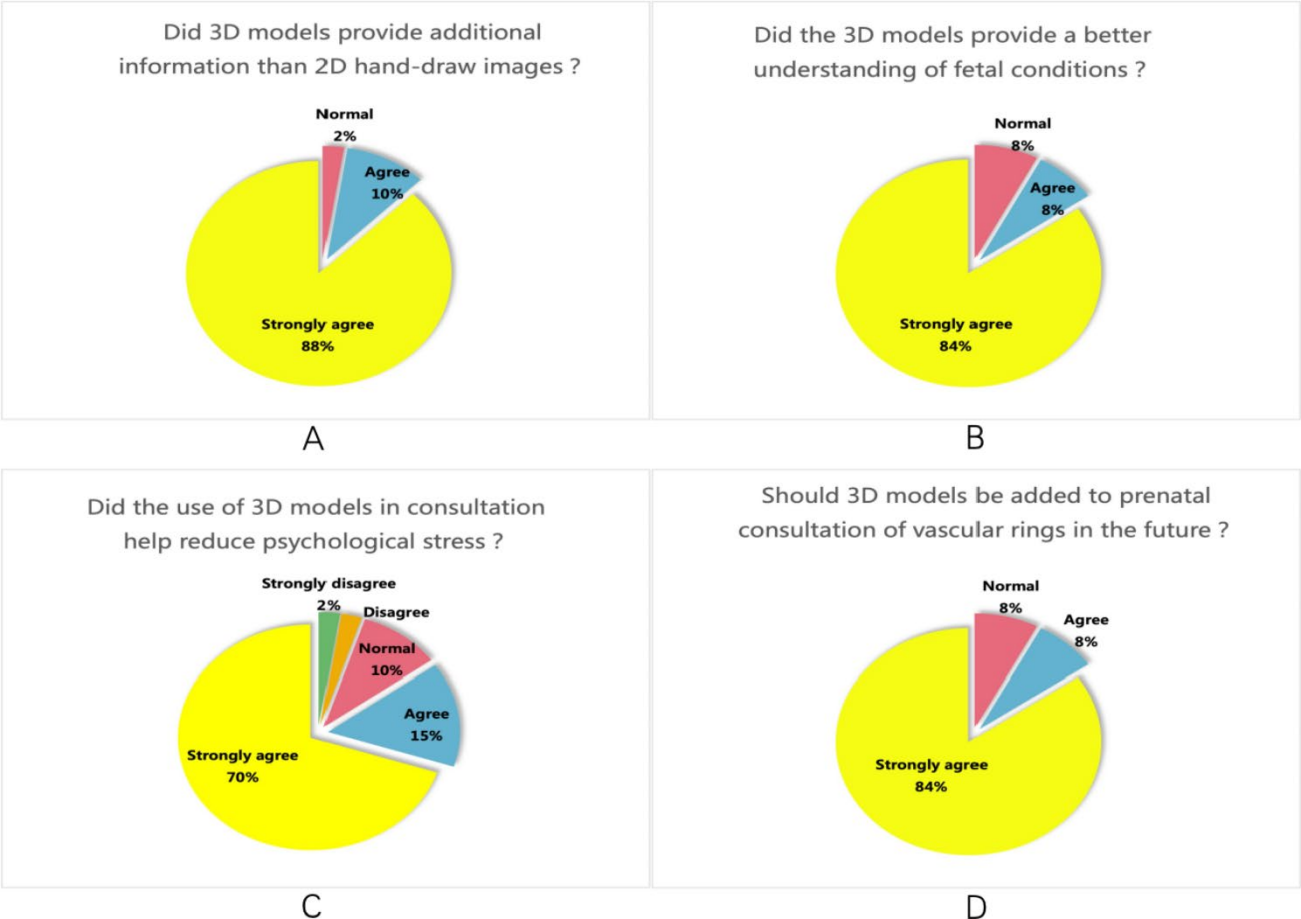
3D Model Questionnaire for Parent. **A.**, Did 3D models provide additional information compared with 2D hand-drawn images? **B.**, Did the 3D models provide a better understanding of foetal conditions? **C.**, Did the use of 3D models in consultation help reduce psychological stress? **D.**, Should 3D models be added to prenatal consultations regarding the diagnosis of vascular rings in the future?

The results of the parental questionnaire showed that 98% of parents agreed that the 3D model could provide more beneficial information than2D hand-drawn schematic diagram, and 92% of parents agreed that the 3D model was helpful to understand the abnormality of the foetal aorta and its branches. 85% of parents agreed that 3D models could help to provide information about the disease in advance and reduce psychological burden. 92% of parents recommended using 3D models in future prenatal consultations (Fig. 7).

## Discussion

VRs refer to a group of congenital vascular anomalies that encircle and compress the oesophagus and trachea [13]. There are many types of VRs. Due to the limitation of the microscopic brachiocephalic blood vessel field of view and sampling angle, it is difficult for traditional ultrasound to display the whole picture regarding the origin and direction of the tortuous branch blood vessels. Thus, some sonographers lack experience and knowledge regarding VRs. Sonographers often exclusively focus on the O- or U-shaped ring around the trachea displayed in the 3VT view, but some types of VRs show the same three-vessel and trachea (3VT) view, which makes it difficult to explain the complex spatial structure of the VRs to inexperienced young physicians and foetal parents.

Three-dimensional printing provides additional spatial information for the prenatal stage [7, 14–16]. With the development of ultrasound technology, Doppler flow graphs in STIC mode can be easily post-processed and printed by 3D printers. However, due to the large number of vessels in the upper mediastinum, the image of small vascular volume is easy to produce aliasing effect. In previous studies, our team preliminarily verified the feasibility of constructing 3D models of foetal hearts and large arteries based on STIC volume data and successfully constructed a series of foetal heart models with abnormal ventricular artery connections [11]. In this study, we further focused on the great arterial branches of the foetal superior mediastinum, through semi-automatic segmentation of the original STIC volume image, layer by layer tracking, each anatomical structure can be used to accurately locate and segment into a single entity to avoid overlap. Furthermore, by manually selecting the structure of the desired regions of interest (ROIs), any anatomy that does not required to be printed can be eliminated, leaving only the specific blood vessels to be printed, obtaining different types of VR models. Through in-depth observation of the anatomical structure and spatial relationship of the great arteries, we hope to provide new technical imaging support to explain the structural complexity of VRs. We also seek to evaluate the feasibility of the use of 3D models in educating medical staff about prenatal VR diagnoses and counselling parents of foetuses with VRs.

Given the complexity of congenital heart disease (CHD), it is difficult to explain the complex 3D cardiac anatomy using simple 2D diagrams, as the true spatial relationships of cardiac structures are not represented [17]. It has been demonstrated that individuals vary in their ability to deduce 3D spatial relationships from 2D diagrams [18]. The inclusion of 3D printed models in CHD educational sessions thereby removes the component of 2D image interpretation and “levels the playing field” for all learners [17]. In addition, 3D-printed adult or paediatric heart models have been shown to serve as a novel teaching tool in medical education and training [19–23]. Our research shows that this novel model teaching tool is also suitable for teaching about prenatal VRs. The feedback forms also showed that residents found the models to be useful and engaging. The scores for learning interest, learning efficiency, educational depth, novelty, inspiring, and overall satisfaction of the teaching method were all greater in the 3D group compared with the 2D group, and the students’ objective performance in VRs postlecture scores was significantly improved. These results are encouraging and confirm the anatomical advantages of using 3D printing. To the best of our knowledge, this is the first time that 3D printing technology has been used to visualize vessels in the foetal upper mediastinum. The 3D model provides anatomical details of the origin and spatial course of major arteries and their branches, and the vascular ring formed around the trachea is also visually clear. The models had vivid effects and created vivid three-dimensional senses, providing an effective tool to help teach complex VR lesions. Furthermore, allowing hands-on learning with physical models inherently increases the interactivity of a session, potentially increasing learners’ retention of knowledge regarding CHD [20]. Although this study did not specifically assess long-term knowledge retention, learner satisfaction differed significantly in the 3D model group compared with the 2D model group and is known to correlate with the retention of knowledge by adult learners in other domains [24]. Long-term knowledge retention will be formally assessed in future studies.

The same benefits that apply to medical education can apply to parental counselling. In addition to the accurate diagnosis of congenital vascular rings during the prenatal period, effective parent consultation is also critical. Classical visual education materials include 2D drawings, which can be very difficult to understand since the cardiac and vessel regions exhibit abnormal anatomic features. Additionally, parents often experience additional anxiety and stress during counselling that further impacts knowledge exchange [25]. Studies have shown that 3D-printed models can provide a new medium for communication with parents of children with congenital heart disease [26]. 3D models are considered to be more direct and easier to use than medical images (such as echocardiography), and the use of 3D printed models as visual educational materials may greatly improve parent counselling [5, 27–29]. Similarly, the 3D model can also be used to improve future parents’ understanding and ability to cope with the condition of the VRs in the foetus. The customized information provided by the model can provide families with sufficient time to gather resources, make decisions and prepare for a reasonable postpartum management approach during birth in advance [30]. Our preliminary research demonstrates that parents respond positively and enthusiastically to the use of 3D models compared to traditional 2D images. In addition to helping parents to better understand their children’s conditions, the models can also help reduce psychological stress. Parents generally agree that 3D-printed models are a valuable tool in prenatal consultations for congenital vascular rings and hope to include such models in future consultations.

Although this is the first study to quantitatively evaluate the use of 3D models in medical education and prenatal counselling in the prenatal stage, our sample size may not be sufficient to assess the true impact of 3D printing on medical education and prenatal counselling. We need to expand the sample size and refine the objective assessments of its true impact. It is also important to note that although 3D printing has shown promise in the current study, the promotion of this new technology remains limited. The general consensus is that 3D printing requires significant additional time, and operators must be familiar with foetal cardiac anatomy and proficient in ultrasound techniques and software operation skills [31–33]. After the research team acquires a certain amount of experience in postprocessing operations, it still takes 4 to 5 h to complete a printed model. It is hoped that the combination of artificial intelligence and machine learning algorithms can be applied to the image postprocessing process, thereby improving the efficiency of image acquisition in the 3D printing process. In addition, current prenatal 3D printing research shows that the models are all rigid and inelastic anatomical models. Future work may require developing models with tactile accuracy and stable performance and improving current 3D printed models by colour-coding different parts to enhance patient and student understanding to provide more effective tools for teaching and prenatal counselling.

## Conclusions

Three-dimensional printing technology allows extracorporeal reconstruction of foetal superior mediastinal vessels. Three-dimensional models can accurately depict the spatial anatomy of the foetal aortic arch and its branches, providing a new tool for displaying different types of vascular rings in the prenatal stage. This tool helps medical students and families understand the complex structure of foetal great vessels, which can positively impact medical teaching and prenatal counselling.

## Data Availability

The datasets supporting the conclusions of this article are included within the manuscript. The authors would like to share raw anonymized video data related to the current study, which could only be used for personal study. The demanders may contact the corresponding author.

## Abbreviations

3D: Three-dimensional
2D: Two-dimensional
3DUS: Three-dimensional ultrasound
AO: Aorta
CHD: Congenital heart disease
DA: Ductus arteriosus
LPA: Left pulmonary artery
PA: Pulmonary artery
RPA: Right pulmonary artery
STIC: Spatio-temporal image correlation
STL: Standard tessellation language
VR: Vascular ring

## References

[1] Abundo MA, Pang RK (1967). Complete vascular ring as a cause of esophageal and tracheal compression. Review of the literature and report of a case. Hawaii Med J.

[2] Ruzmetov M, Vijay P, Rodefeld MD, Turrentine MW, Brown JW (2009). Follow-up of surgical correction of aortic arch anomalies causing tracheoesophageal compression: a 38-year single institution experience. J Pediatr Surg.

[3] Evans WN, Acherman RJ, Ciccolo ML, Carrillo SA, Mayman GA, Luna CF, Rollins RC, Castillo WJ, Restrepo H (2016). Vascular Ring diagnosis and management: notable Trends over 25 years. World J Pediatr Congenit Heart Surg.

[4] D’Antonio F, Khalil A, Zidere V, Carvalho JS (2016). Fetuses with right aortic arch: a multicenter cohort study and meta-analysis. Ultrasound Obstet Gynecol.

[5] Awori J, Friedman SD, Chan T, Howard C, Seslar S, Soriano BD, Buddhe S. 3D models improve understanding of congenital heart disease.3D Print Med. 2021;7:26. doi: 10.1186/s41205-021-00115-7. 10.1186/s41205-021-00115-7 PMC8411549 34471999

[6] Merz E, Pashaj S (2017). Advantages of 3D ultrasound in the assessment of fetal abnormalities. J Perinat Med.

[7] Liang J, Ma Q, Zhao X, Pan G, Zhang G, Zhu B, Xue Y, Li D, Lu B (2022). Feasibility analysis of 3D Printing with prenatal Ultrasound for the diagnosis of fetal abnormalities. J Ultrasound Med.

[8] Miller JL, Ahn ES, Garcia JR, Miller GT, Satin AJ, Baschat AA (2018). Ultrasound-based three-dimensional printed medical model for multispecialty team surgical rehearsal prior to fetoscopic myelomeningocele repair. Ultrasound Obstet Gynecol.

[9] Jarvis D, Griffiths PD, Majewski C (2016). Demonstration of normal and abnormal fetal brains using 3D Printing from in Utero MR Imaging Data. AJNR Am J Neuroradiol.

[10] Werner H, Lopes J, Tonni G, Araujo Junior E (2016). Maternal fetal attachment in blind women using physical model from three dimensional ultrasound and magnetic resonance scan data: six serious cases. J Matern Fetal Neonatal Med.

[11] Huang J, Shi H, Chen Q, Hu J, Zhang Y, Song H, Zhou Q (2021). Three-dimensional printed model fabrication and effectiveness evaluation in fetuses with congenital heart disease or with a normal heart. J Ultrasound Med.

[12] Arazińska A, Polguj M, Szymczyk K, Kaczmarska M, Trębiński Ł, Stefańczyk L (2017). Right aortic arch analysis-anatomical variant or serious vascular defect?. BMC Cardiovasc Disord.

[13] Yoo SJ, Lee YH, Kim ES, Ryu HM, Kim MY, Choi HK, Cho KS, Kim A (1997). Three-vessel view of the fetal upper mediastinum: an easy means of detecting abnormalities of the ventricular outflow tracts and great arteries during obstetric screening. Ultrasound Obstet Gynecol.

[14] Chen SA, Ong CS, Hibino N, Baschat AA, Garcia JR, Miller JL (2018). 3D printing of fetal heart using 3D ultrasound imaging data. Ultrasound Obstet Gynecol.

[15] Guo YT, Hou N, Liang JH, Zhang ZK, Cao TS, Yuan LJ (2020). Three-dimensional printed multicolor normal and abnormal fetal hearts based on ultrasound imaging data. Ultrasound Obstet Gynecol.

[16] VanKoevering KK, Morrison RJ, Prabhu SP, Torres MF, Mychaliska GB, Treadwell MC, Hollister SJ, Green GE (2015). Antenatal Three-Dimensional Printing of aberrant facial anatomy. Pediatrics.

[17] Smerling J, Marboe CC, Lefkowitch JH, Pavlicova M, Bacha E, Einstein AJ, Naka Y, Glickstein J, Farooqi KM (2019). Utility of 3D printed Cardiac Models for Medical Student Education in congenital heart disease: across a spectrum of Disease Severity. Pediatr Cardiol.

[18] Hoyek N, Collet C, Rastello O, Fargier P, Thiriet P, Guillot A (2009). Enhancement of mental rotation abilities and its effect on anatomy learning. Teach Learn Med.

[19] McMenamin PG, Quayle MR, McHenry CR, Adams JW (2014). The production of anatomical teaching resources using three-dimensional (3D) printing technology. Anat Sci Educ.

[20] Loke YH, Harahsheh AS, Krieger A, Olivieri LJ (2017). Usage of 3D models of tetralogy of Fallot for medical education: impact on learning congenital heart disease. BMC Med Educ.

[21] SuW, Xiao Y, He S, Huang P, Deng X (2018). Three-dimensional printing models in congenital heart disease education for medical students: a controlled comparative study. BMC Med Educ.

[22] White SC, Sedler J, Jones TW, Seckeler M (2018). Utility of three-dimensional models in resident education on simple and complex intracardiac congenital heart defects. Congenit Heart Dis.

[23] Lim KHA, Loo ZY, Goldie SJ, Adams JW, McMenamin PG (2016). Use of 3D printed models in medical education: a randomized control trial comparing 3D prints versus cadaveric materials for learning external cardiac anatomy. Anat Sci Educ.

[24] Yammine K, Violato C (2016). The effectiveness of physical models in teaching anatomy: a meta-analysis of comparative studies. Adv Health Sci Educ Theory Pract.

[25] Biglino G, Capelli C, Wray J, Schievano S, Leaver LK, Khambadkone S, Giardini A, Derrick G, Jones A, Taylor AM. 3D-manufactured patient-specific models of congenital heart defects for communication in clinical practice: feasibility and acceptability. BMJ Open 2015 30;5:e007165. doi: 10.1136/bmjopen-2014-007165. 10.1136/bmjopen-2014-007165 PMC4420970 25933810

[26] Schlund M, Levaillant JM, Nicot R (2020). Three-Dimensional Printing of prenatal Ultrasonographic diagnosis of cleft lip and palate: presenting the needed “Know-How” and discussing its use in parental education. Cleft Palate Craniofac J.

[27] Biglino G, Capelli C, Wray J, Schievano S, Leaver LK, Khambadkone S, Giardini A, Derrick G, Jones A, Taylor AM (2015). 3D-manufactured patient-specific models of congenital heart defects for communication in clinical practice: feasibility and acceptability. BMJ Open.

[28] Lau IWW, Liu D, Xu L, Fan Z, Sun Z (2018). Clinical value of patient-specific three-dimensional printing of congenital heart disease: quantitative and qualitative assessments. PLoS ONE.

[29] Biglino G, Moharem-Elgamal S, Lee M, Tulloh R, Caputo M (2017). The perception of a three-dimensional-printed heart model from the perspective of different stakeholders: a Complex Case of Truncus Arteriosus. Front Pediatr.

[30] Li TG, Li QL, Ma B, Qi PA, Wang J, Yang L (2021). Prenatal diagnosis of complete vascular ring using high-definition flow render mode and spatiotemporal image correlation. Echocardiography.

[31] Sun Z, Lau I, Wong YH, Yeong CH (2019). Personalized three-dimensional printed models in congenital heart disease. J Clin Med.

[32] Anwar S, Singh GK, Miller J, Sharma M, Manning P, Billadello JJ, Eghtesady P, Woodard PK (2018). 3D Printing is a transformative technology in congenital heart disease. JACC Basic Transl Sci.

[33] Yoo SJ, Hussein N, Peel B, Coles J, van Arsdell GS, Honjo O, Haller C, Lam CZ, Seed M, Barron D (2021). 3D modeling and Printing in congenital heart surgery: entering the stage of Maturation. Front Pediatr.

